# Biochemical, Molecular, and Clinical Characterization of Patients With Primary Carnitine Deficiency via Large-Scale Newborn Screening in Xuzhou Area

**DOI:** 10.3389/fped.2019.00050

**Published:** 2019-02-26

**Authors:** Wei Zhou, Huizhong Li, Ting Huang, Yan Zhang, Chuanxia Wang, Maosheng Gu

**Affiliations:** Xuzhou Maternity and Child Health Care Hospital, Xuzhou, China

**Keywords:** primary carnitine deficiency (PCD), *SLC22A5* gene, newborn screening, tandem mass spectrometry (MS/MS), maternal PCD

## Abstract

**Background:** Primary carnitine deficiency (PCD) is attributed to a variation in the *SLC22A5* (OCTN2) gene which encodes the key protein of the carnitine cycle, the OCTN2 carnitine transporter. PCD is typically identified in childhood by either hypoketotic hypoglycemia, or skeletal and cardiac myopathy. The aim of this study was to the clinical, biochemical, and molecular characteristics of PCD patients via newborn screening with tandem mass spectrometry (MS/MS).

**Methods:** MS/MS was performed to screen newborns for inherited metabolic diseases. *SLC22A5* gene mutations were detected in the individual and/or their family member by DNA mass array and next-generation sequencing (NGS).

**Results:** Among the 236,368 newborns tested, ten exhibited PCD, and six others were diagnosed with low carnitine levels caused by their mothers, who had asymptomatic PCD. The incidence of PCD in the Xuzhou area is ~1:23,637. The mean initial free carnitine (C_0_) concentration of patients was 6.41 ± 2.01 μmol/L, and the follow-up screening concentration was 5.80 ± 1.29 μmol/L. After treatment, the concentration increased to 22.8 ± 4.13 μmol/L.

**Conclusion:** This study demonstrates the important clinical value of combining MS/MS and NGS for the diagnosis of PCD and provides new insight into the diagnosis of PCD and maternal patients with PCD using C_0_ concentration and *SLC22A5* mutations.

## Introduction

Primary carnitine deficiency (PCD), a of carnitine cycle disorder, represents an autosomal recessive defect that occurs on the *SLC22A5* (OCTN2) gene (1, 2). The organic cation transporter (OCTN) family is essential for transporting organic cation compounds. One member of this family, OCTN2, which is encoded by the *SLC22A5* gene, can transfer carnitine across the cell membrane (3). Without the ability to transport carnitine into the cell, long-chain fatty acids, which are participated in fatty acid β-oxidation, cannot enter the mitochondrial matrix and provide energy for the body (4). As one of the most common fatty acid oxidation and metabolic diseases, PCD was first detected by measuring plasma free carnitine (C_0_) levels in 1988 (5, 6). However, the connection between PCD and mutations located on the *SLC22A5* gene was not demonstrated until 10 years later (1, 2).

The incidence rate of PCD is approximately 1:40,000 in Japan, but the incidence is 1:297 in the Faroe Islands, which is extremely high (7, 8). In Taiwan, the incidence of PCD is 1:18,543, which is similar to that in the Xuzhou area, which has a current prevalence of 1: 23,637 (9).

The most typical form of PCD is characterized by progressive infantile-onset cardiomyopathy, with weakness, peripheral neuropathy, and recurrent hypoglycemic hypoketotic encephalopathy. Cardiomyopathy, skeletal muscle weakness, and mildly elevated creatine kinase levels occasionally occur, which have a considerable impact on metabolic decompensation (10, 11). The occurrence of death due to cardiac failure before diagnosis suggests that PCD can be fatal without treatment. Some infants of asymptomatic maternal patients with PCD have been reported to have low C_0_ levels, which emphasize the necessity of universal newborn screening, including the screening of asymptomatic newborns and their mothers (12). Infants with low levels of C_0_ were screened for PCD via newborn screening programs through tandem mass spectrometry (MS/MS) (13, 14). With the application of MS/MS in neonatal screening, increasing numbers of children in our area are examined, diagnosed and treated early, and the prognosis has been favorable.

Here, ten patients with PCD were screened by MS/MS and diagnosed by DNA sequence analysis, while six infants whose mothers were diagnosed with PCD exhibited low C_0_ levels at the first neonatal screening. The clinical, biochemical, and molecular characteristics of the six infants were subsequent analyzed. In addition, two other families were conducted PCD, and the younger family members presented low C_0_ levels.

## Patients and Methods

### Study Population

From November 2015 to December 2017, 236,368 newborns were recruited for PCD screening at the Genetic Medicine Center in Xuzhou Maternity and Child Health Care Hospital. C_0_ levels were measured by MS/MS, with a dried spot of 2 mg/dL (134 μmol/L) whole blood that was collected from the infants' plantar surface 48–72 h after birth. Subsequently, the NeoBase Non-derivatized MSMS Kit (PerkinElmer, Finland) was used for prepreparation of samples at room temperature (21–24°C) and an appropriate humidity (50–70%). Several instrumental parameters of the MS/MS are as follows: ion mode-electrospray^−^/MS/MS; source temperature-120°C/desolvation temperature-350°C. Informed consent was issued by the guardians of all the patients before clinical testing.

### MS/MS Analysis and Diagnostic Criteria

The plasma C_0_ concentration was quantified by MS/MS, with a cut-off value of 2 mg/dL (134 μmol/L) in the whole blood. The cut-off value of C_0_ was 9.63–54 μmol/L.

Patients were diagnosed with PCD based on the following criteria (4):

1. Newborns with a C_0_ value <9.63 μmol/L were rescreened; if the C_0_ value was <5.0 μmol/L in the second screening, the newborn was diagnosed with PCD, and the mothers' C_0_ levels were tested to exclude maternal carnitine deficiency.
2. Newborns with persistent low C_0_ levels (normal>9.63 μmol/L) were readmitted for further diagnosis.
3. Two pathogenic mutations were detected in the *SLC22A5* gene of the OCTN; one mutation was derived from the father and the other was derived from the mother (4).

### DNA Sequence Analysis, Treatment, and Follow-Up

Infants and/or their family members who had abnormal results from the PCD screening provided blood samples, which were sent to Bioscan Genomics, Hangzhou. Subsequently, the targeted DNA sequence was mapped and analyzed by comparing the DNA sequence with a genetic diagnosis panel of hereditary metabolic diseases covering 51 diseases and 98 genes. One of the panels included 16 fatty acid metabolism diseases and 21 genes, including *SLC22A5*. Peripheral blood samples from the patients were used to extract genomic DNA with the omega Genomic DNA Extraction Kit (omega Biotech, USA), and genealogies were determined via Sanger sequencing.

All patients, including those with maternal PCD, were asymptomatic when diagnosed. After diagnosis, 100–300 mg/kg/day of L-carnitine was granted orally 3–4 times per day. The patients were monitored in the clinic once every 2–3 weeks during the initial treatment and then once every 3 months thereafter or a little longer after serum levels of C_0_ stabilized and returned to normal. A normal serum level of C_0_ is approximately 20 μmol/L. C_0_ is considered a reliable marker during treatment, and after treatment, the levels of a series of other acylcarnitines were found to be normal (15). The youngest infant with PCD was monitored for 2.3 years after birth.

### Bioinformatics and Statistical Analysis

Fifty amino acid sequences of *SLC22A5* were downloaded from the NCBI database. Then, sequence logo of *SLC22A5* was built by with WebLogo (16). The point mutation sites in this study were well-conserved in the wild-type (*SLC22*A5). Electrostatic interactions played a crucial role in the process of protein function. To investigate changes in electrostatic properties caused by mutations, adaptive Poisson-Boltzmann solver (APBS) and PDB2PQR were applied to each mutant and wild-type (17). The pqr file of each structure was generated using the PDB2PQR program. The dx file of each structure was generated by utilizing APBS. The pqr file and dx file were then uploaded in VMD to show the molecular surface electrostatic potential map (18). High-quality 3-D images of the protein were drawn by PyMOL (19).

SPSS 16.0 statistical software package was used (SPSS Inc., Chicago, IL,USA). Logistic regression was performed with the 200,000 healthy newborns in Xuzhou. A confidence interval of 0.5–99.5% was selected as standard clinic reference interval.

## Results

### Clinical and Biochemical Description

**Figure 1:**
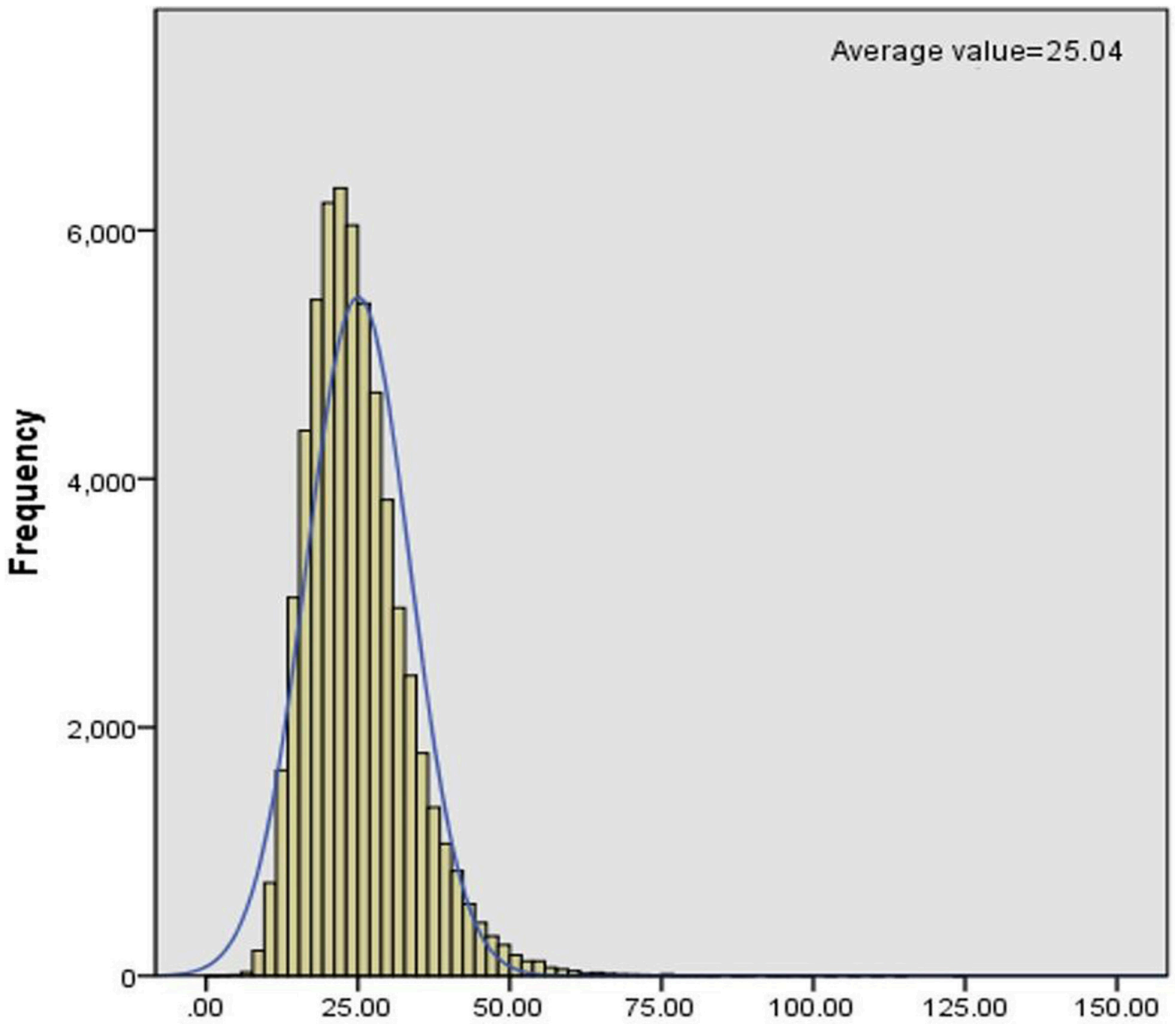
Frequency distribution histogram of C_0_.

C_0_ levels in dry blood spots were detected through MS/MS. We collected clinical data of PCD patients from November 2015 to December 2017 and then analyzed the levels of C_0_, which was described as a normal distribution (Figure 1). Statistically, the reference interval was in line with the percentiles of the 200,000 health screening samples in Xuzhou. We chose a confidence interval of 0.5–99.5%, [CI]: 9.65–54.59. As a consequence, the reference range of C_0_ in clinic was 9.65–54.59 μmol/L in Xuzhou.

**Figure 2:**
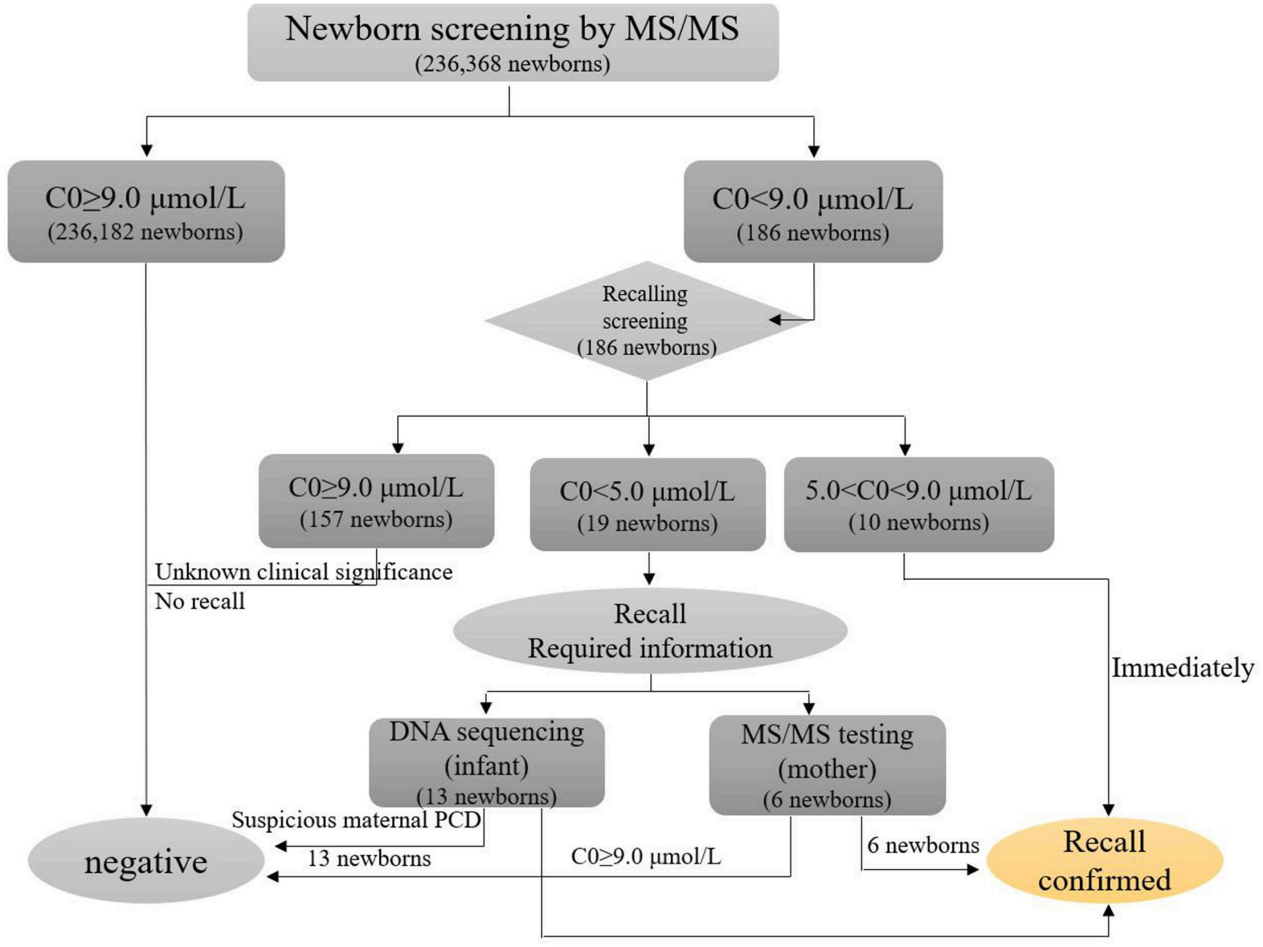
Flow diagram for PCD screening and follow-up.

Among the 236,368 newborns screened by MS/MS, 186 infants initially exhibited C_0_ levels that were less than the normal range. In addition, the PCD screening protocol has been modified in daily practice, as showed in Figure 2. Out of 186 infants that were suspected to have PCD from the neonatal screening, 16 of the 186 cases were confirmed to have *SLC22A5* mutations. Ten newborns were diagnosed with PCD, and another six individuals were diagnosed with maternal PCD (theoretically 1 in 23,637 in our region). However, data on PCD incidence are limited and insufficient, which is partially attributed to asymptomatic individuals. To evaluate the incidence of PCD, further work should be performed. Ten PCD patients included eight males and two females; the PCD patients were full-term children with normal birth weight, with the exception of Case 7 and had no clinical symptoms. The clinical and biochemical characteristics of the ten patients with PCD are showed in Table 1. The infant in Case 9 had a 12-year-old sister, and the infant in Case 10 had a twin-brother. Neither patient was deemed to have any significant medical history. The Case 9 infant's sister had the same genetic mutations as the Case 9 infant; however, the Case 10 infant's brother had a normal serum carnitine level.

**Table 1:** Clinical, biochemical, and molecular characteristics of the ten cases of children with PCD.

**Case**	**Current age**	**Sex**	**Birth weight (kg)**	**Gestational weeks**	**C**_****0****_ **level**, **μmol/L**		***SLC22A5*** **gene mutation**	
					**Primary screening**	**After treatment(mean)**	**Allele 1 (maternal)**	**Allele 2 (paternal)**
1	2y4m	M	3.00	40	6.74	30.23	c.1400C > G, p. S467C	c.1400C > G, p. S467C
2	1y5m	M	3.10	38	4.02	56.60	c.1400C > z G, p. S467C	c.1400C > G, p. S467C
3	1y3m	F	3.25	39 + 4	6.36	42.81	c.1093A > C, p. T365P	c.1400C > G, p. S467C
4	1y2m	M	3.40	39 + 1	7.05	3.71	c.428C > T, p. P143L	c.1400C > G, p. S467C
5	9m	M	3.50	39 + 4	7.36	36.94	c.1400C > G, p. S467C	c.1400C > G, p. S467C
6	7m	F	3.00	39 + 5	7.18	32.75	c.1400C > G, p. S467C	c.1400C > G, p. S467C
7	7m	M	2.50	33 + 2	7.82	-	c.761G > A, p. R254Q	c.865C > T, p. R289^*^
8								
Infant	1y7m	M	3.20	41 + 1	7.80	26.78	c.1195C > T, p. R399W	c.1400C > G, p. S467C
Mother					18.56	-	c.1195C > T, p. R399W	-
Father							-	c.1400C > G, p. S467C
9								
Infant	1y3m	M	3.30	39 + 2	6.89	15.01	c.1400C > G, p. S467C	c.92C > T, p. P31L
Mother					21.09	-	c.1400C > G, p. S467C	-
Father							NT	
Sister	12y7m	F	3.00	39	3.25	11.64	c.1400C > G, p. S467C	c.92C > T, p. P31L
10								
Infant	7m	M	3.60	41 + 2	7.65	14.56	c.1400C > G, p. S467C	c.1195C > T, p. R399W
Mother					15.45	-	c.1400C > G, p. S467C	-
Father							NT	
Brother		M	3.50	41 + 2	11.46	-	NT	

*F, Female; M, Male; y, year; m, month; NT, not tested*.

*, *nonsense mutation*.

### Clinical Characteristics of Maternal PCD

In six families, the infants were identified to be free of PCD; conversely, their mothers were diagnosed. The six families came from different counties in Xuzhou; Families 1 and 3 were from Peixian, Family 2 was from Suining, Family 4 was from Xinyi and the last two families were from Fengxian. All six newborns were normal-birth-weight infants who had unremarkable neonatal physical examinations. All births were by eutocia, and none of the mothers had complications or discomfort. The newborn screening of each infant was performed within 7 days after birth, and if the infant exhibited a low C_0_ value, the value was verified by a subsequent plasma carnitine analysis (Table 2). Subsequently, all individuals received carnitine supplementation at a dose of 100–300 mg/kg/day. After treatment with L-carnitine, follow-up carnitine profiles showed normal or elevated carnitine levels in all infants. The recent follow-up monitoring of C_0_ levels in the six infants took place at the ages of 1, 3, 3, 2, 2, and 3 months. All of the infants were asymptomatic and were developing age appropriately.

**Table 2:** Plasma carnitine and *SLC22A5* gene sequencing results for reported maternal PCD.

	**Newborn screening free carnitine, μmol/L 9.63-54**	**Plasma carnitine**, **μmol/L**		***SLC22A5*** **gene sequencing**	
		**Timing relative to carnitine supplementation**	**Free carnitine(C_**0**_) 9.63-54**	**Allele 1**	**Allele 2**
**Family 1**					
Infant	7.04	Before	7.04	c.1400C > G, p. S467C	–
		6 days after	8.47		
Mother		Before	1.98	c.1400C > G, p. S467C	c.1400C > G, p. S467C
		After	-		
**Family 2**					
Infant	5.61	Before	13.28	NT	
		2 weeks after	16.84		
Mother		Before	5.66	c.797C > T, p. P266L	c.1400C > G, p. S467C
		6 weeks after	20.37		
**Family 3**					
Infant	5.85	Before	3.72	c.95A > G, p. N32S	-
		2 months after	48.23		
Mother		Before	1.73	c.95A > G, p. N32S	c.1400C > G, p. S467C
		2 months after	4.10		
**Family 4**					
Infant	3.20	Before	12.49	c.1400C > G, p. S467C	-
		2 months after	20.32		
Mother		Before	3.62	c.1400C > G, p. S467C	c.1400C > G, p. S467C
		9 weeks after	16.12		
**Family 5**					
Infant	5.90	Before	4.93	c.1400C > G, p. S467C	-
		5 weeks after	74.64		
Mother		Before	2.05	c.1400C > G, p. S467C	c.1400C > G, p. S467C
		After	-		
**Family 6**					
Infant	3.96	Before	3.34	c.1400C > G, p. S467C	-
		3 months after	87.03		
Mother		Before	2.73	c.1462C > T, p. R488C	c.1400C > G, p. S467C
		3 months after	16.80		

*NT, not tested*.

Table 2 shows a clear trend of decreasing of plasma carnitine levels in the six mothers. The mothers of Families 1 and 5 were 24 years old, while both the mothers of Families 2 and 4 were 28 years old. The mothers of Families 3 and 6 were 26 and 27 years old, respectively. None of the mothers were reported to have significant medical history. Each infant was their first child. The mothers were given 1–2g of L-carnitine daily. After treatment, C_0_ values of the mothers of Families 2, 4, and 6 increased to within the normal range. However, the mother of Family 3 who did not receive carnitine supplementation sustained a low carnitine level (Table 2). Unfortunately, the mothers of Families 1 and 5 did not follow-up after the initial detection for several reasons.

**Figure 3:**
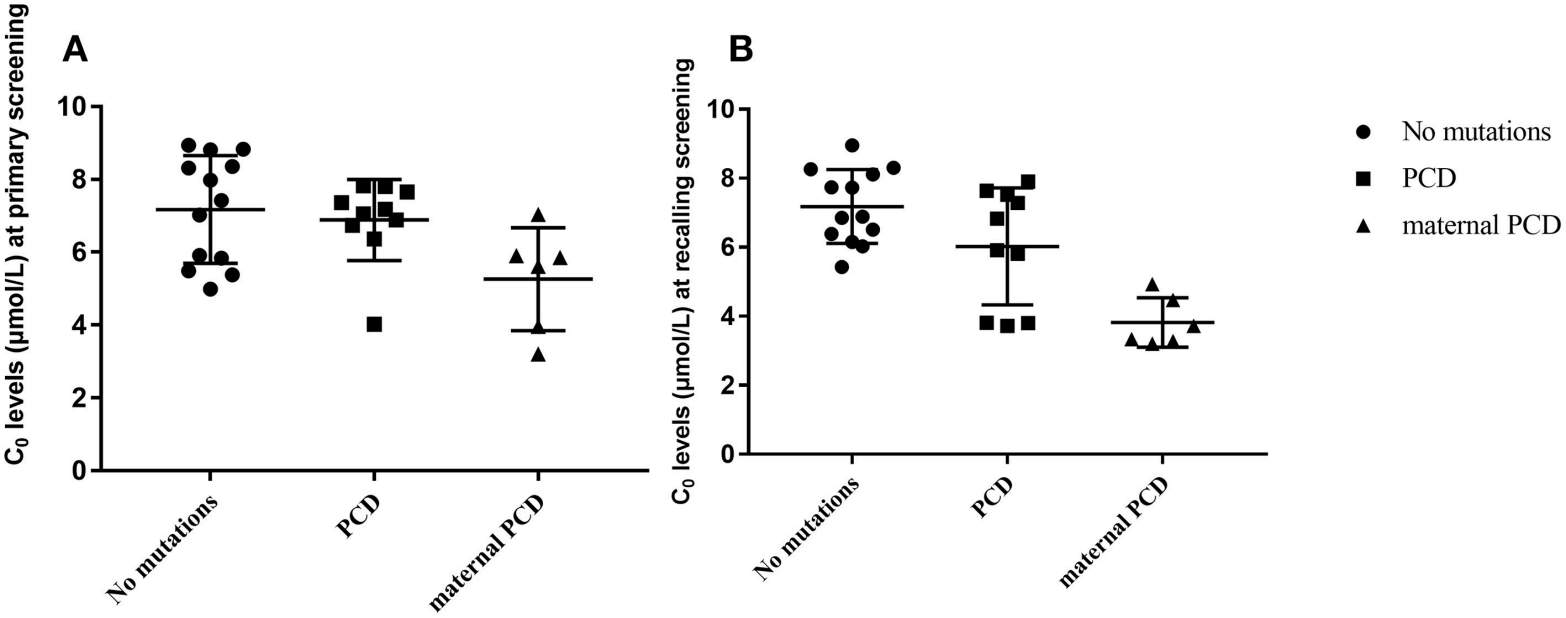
Metabolite measurement in PCD compared to no mutation, PCD and maternal PCD patients. **(A)** Primary screening result of C_0_. **(B)** Recalling screening results of C_0_.

In summary, of the 236,368 newborns screened by MS/MS, 29 (0.012%) had C_0_ levels that were below the cut-off values and were recorded as positive for PCD first the screening. According to the follow-up testing, 10 cases of PCD and 6 cases of maternal PCD, were confirmed and treated in our center. The other 13 cases with no mutations were regarded as negative for PCD in the clinic after a period of monitoring. As showed in Figure 3, C_0_ was primarily low in PCD patients, while the level of C_0_ was not different between healthy and PCD newborns. The serum levels of C_0_ in newborns with maternal PCD were far lower than the levels of other newborns at the follow-up screening, (as showed in Figure 3), but the distribution of the population without mutations was near the normal level. To avoid misdiagnose, further data collection is required due to other rare forms of PCD that can affect the diagnosis. Almost no individuals in the mutation group were followed up, but the results were normal at present.

### *SLC22A5* Gene Sequencing Results

DNA sequencing was further performed in 16 newborns with low C_0_ levels from 16 separate families. The *SLC22A5* gene mutations are described in Tables 1, 2.

**Figure 4:**
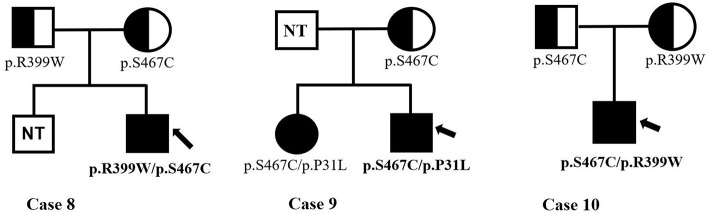
The genetic family map of case 8,9,10. Bold and arrow mark the proband. The mutant types mark under the individuals. NT, not test.

Infants with PCD were shown to have two mutations, one from each of their parents, that is so-called paternal and maternal alleles. The patients in Cases 3, 4, and 9 are being detected with compound heterozygous for one novel missense variant, which was a paternal or maternal allele. The most common mutation of the *SLC22A5* gene in our area is c.1400C > G (p. S467C) mutation. Figure 4 shows the genetic family map of Cases 8, 9, and 10. The infant in Case 8 was compound heterozygous for c.1195C > T (p. R399W) and c.1400C > G (p. S467C) because his mother and father were heterozygous for the missense mutations c.1195C > T (p. R399W) and c.1400C > G (p. S467C), respectively. However, the new mutation type c.92C > T (p. P31L) may have been from a father who was not tested. The Case 10 infant was identified as compound heterozygous for two missense variants c.1195C > T (p. R399W) and c.1400C > G (p. S467C), whereas his mother was a carrier for only the c.1400C > G (p. S467C) variant. Neither the infant's twin-brother nor their father were tested.

In the maternal PCD families, the mothers of Families 1, 4 and 5 were found to be homozygous for a classic missense mutation, c.1400C > G (p. S467C). The infants were carriers of the c.1400C > G (p. S467C) variant. The mother of Family 3 was compound heterozygous for two missense mutations, c. 95A > G (p. N32S) and c.1400C > G (p. S467C). Additionally, the mother of Family 6 was also compound heterozygous for two common missense mutations, c.1462C > T c. (p. R488C) and c.1400C > G (p. S467C). In these two families, carriers were verified with c. 95A > G (p. N32S) or c.1400C > G (p. S467C) mutations. The mother of Family 2 was shown to be compound heterozygous, carrying a novel missense mutation, c.797C > T (p. P266L), and a classified missense mutation, c.1400C > G (p. S467C). The infant in Family 2 was assessed clinically and was found to be free of carnitine deficiency; DNA analysis was not performed, but she was presumed to be an obligate carrier given the diagnosis of her mother.

### The Frequencies and Locations of *SLC22A5* Gene Mutations

**Figure 5:**
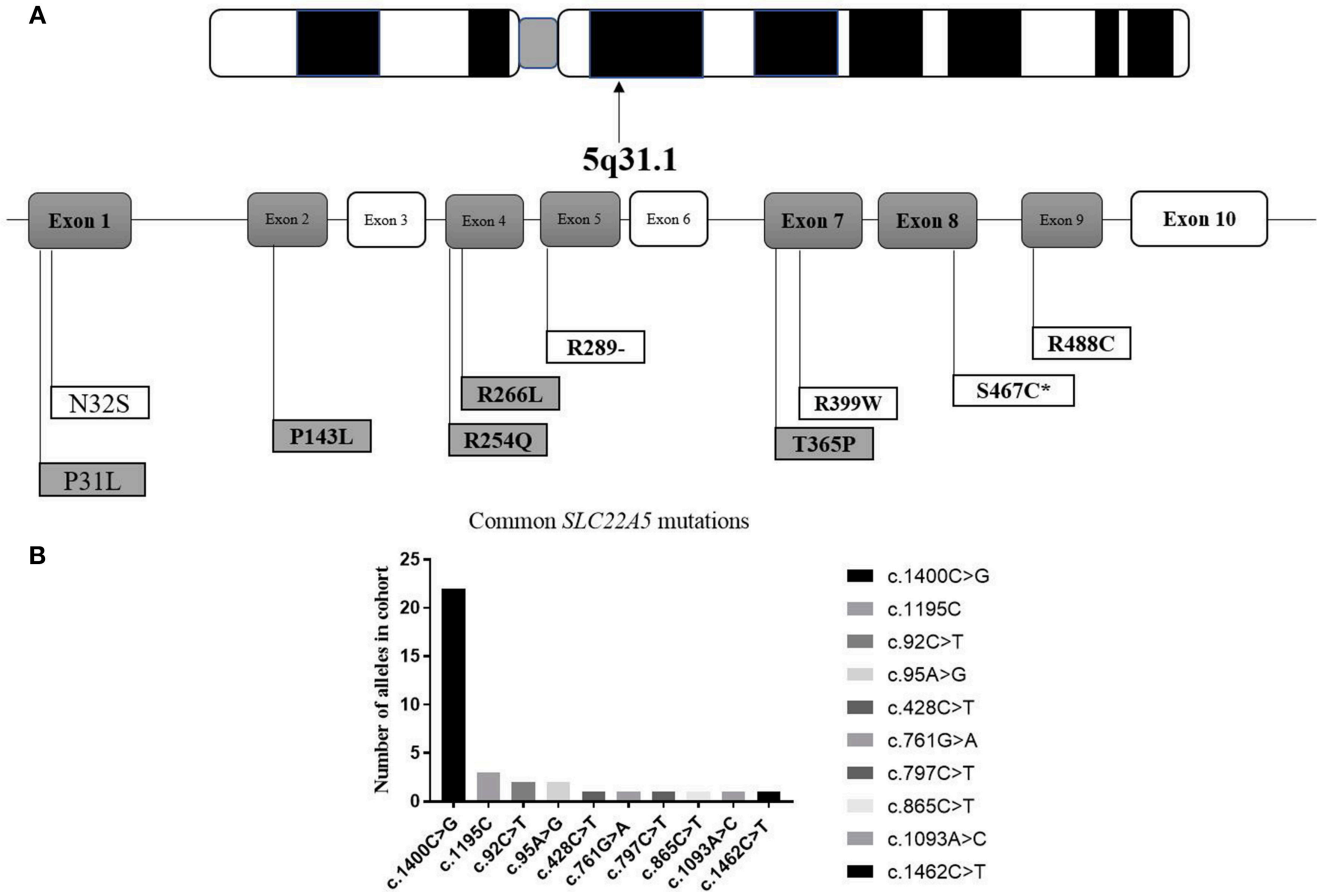
Graphic presentation of mutations/unclassified missense variant identified in our study. **(A)** structure of *SLC22A5* gene. Bold and italic represent novel changes. Gray represents unclassified missense variant and exon. ATPB, ATP binding domain. *mark the most common type of mutation in *SLC22A5 gene*. **(B)** Frequency of *SLC22A5* gene mutations.

It can be observed in the data that a total of 43 mutant alleles with 10 different mutations/unclassified missense variants (Table 3 and Figure 5) were verified in 26 cases (Tables 1, 2). Expect for five classical mutations, the additional mutations were not classified and might be considered unclassified types. Five novel missense variants were considered unclassified mutations. Indeed, previous studies have suffered from methodological limitations and rare cases, which have found it difficult to determine the significance of these mutations. Some statistical analyses cannot be conducted to explain the correlation between the apparent “novel” mutations and their clinical relevance. However, based on the landscapes of the electrostatic potential for the five *SLC22A5* proteins, the point mutations in the wild-type *SLC22A5* protein could affect its surface electrostatic potential which plays an important role in protein-protein interaction. This result suggests that these genetic substitutions probably result in various *SLC22A5* functions as showed in Figure S1. Moreover, further work will be performed in the future. Among the 10 different mutations that were identified, the most frequently occurring mutation was the c.1400C > G (p. S467C) with a frequency of ~72% (31/43). Serine at position 467 is revolutionaries conserved, as the amino acid change in p. S467C can affect the transmembrane domain 11 (TM11) of the OCTN2 protein, resulting in PCD. Seven cases were homozygous for the constant mutations of c.1400C>G. Seventeen cases, including the 7 homozygous cases, had 2 mutations; 6 cases carried 1 unclassified variant and 1classified mutation; and other cases harbored two classified mutations. Hence, at least one mutation was detected in each person analyzed. Not only were classical mutations identified but unclassified missense variants in all exons were also identified in our area. However, Table 3 is shown that more than 72% of mutant alleles (31/43) were located in exon 8 while the most frequently affected domain was TM11 of the OCTN2 protein.

**Table 3:** Detected mutations and unclassified variants, and their frequencies and locations.

**Mutations/unclassified missense variants**	**Freq**	**Exons**	**Domains**
**c.92C > T (p. P31L)**	2	Exon 1	TM 1
c.95A > G (p. N32S)	2		
c.428C > T (p. P143L)	1	Exon 2	TM 2
**c.761G > A (p. R254Q)**	1	Exon 4	L 5
**c.797C > T (p. P266L)**	1		TM 6
c.865C > T (p. R289^*^)	1	Exon 5	L 6
**c.1093A > C (p. T365P)**	1	Exon 7	TM 7
c.1195C (p. R399W)	3		L 8
c.1400C > G (p. S467C)	22	Exon 8	TM 11
c.1462C > T (p. R488C)	1	Exon 9	L 11
Total	35		

*Bold novel changes in this study; Gray, unclassified missense variant. TM, transmembrane domain. L, inter-transmembrane loop*.

*, *nonsense mutation*.

## Discussion

As indicated in the literature review, PCD is a disease caused by a carnitine absorption barrier. Unfortunately, some significant clinical consequences, even death, can occur if PCD is not treated on time. However, PCD can be dramatically improved with carnitine supplementation. Previous investigations have not presented any exact data about the nationwide prevalence of PCD in China. It has been reported that the incidence of PCD was approximately 1:45,000 in Shanghai (20, 21), approximately 1:22,384 in Zhejiang Province (22) and 1:8,938 in Nanjing, Jiangsu Province (4). In this study, 236,368 newborns were screened and ten PCD cases in infants and six maternal PCD cases were identified by MS/MS from 2015 to 2017 in Xuzhou, China. Thus, the incidence of PCD in the Xuzhou area was 1:23,637, which was similar to that of Zhejiang Province.

The *SLC22A5* gene contains 10 exons and 3 introns located on chromosome 5q31.1. To date, over 110 mutations in the *SLC22A5* gene have been associated with PCD, while c.1400C>G (p. S467C) is considered to be the most common mutation among Chinese patients. Subsequently, 7 pathogenic homozygous mutations were detected in 16 patients and other heterozygous mutations were also noted. This result is in accordance with the findings of earlier reports indicating that the frequency of the c.1400C > G (p. S467C) mutation was as high as 72% (31/43). Unfortunately, what is not yet clear is the prevalence of PCD false-negative assessments in the primary screening in our area. To account for the technical challenge, a rapid novel 2nd-tier test application might reduce the false-negative rate in the clinical setting.

Traditionally, serum C_0_ levels have been assessed (23, 24) based on quantifiable biochemical manifestations and DNA analysis. Sixteen patients showed a lower C_0_ level (<9.63 μmol/L) at the primary screening; with a mean value of 6.48 μmol/L, emphasizing the utility of MS/MS neonatal screening for PCD diagnosis. Extremely low levels of C_0_ were observed in these individuals at the second screening, which seems to be consistent with previous findings that showed that PCD patients often exhibit low C_0_ levels at the follow-up screening. Asymptomatic carriers are individuals who are identified with heterozygous mutations without clinical consequences due to half-normal carnitine transport in their fibroblasts. Despite slightly lower C_0_ levels, these infants had no impact to the degree of the PCD patients. Collectively, plasma carnitine levels alone are not a reliable indicator of a PCD diagnosis, and DNA analysis should also be taken into account.

C_0_ levels of infants reflect their mothers' carnitine levels shortly after birth, because carnitine is provided by the placental tissue matrix to the fetus during intrauterine life (25). Therefore, one reason infant present with low C_0_ levels at the initial screening is that their mothers have PCD. These results reinforced several notions that are in charge of identifying some maternal inborn errors of metabolism (26, 27). To decrease false-positive or false-negative diagnoses, we should also pay more attention to maternal evaluation after the identification of a low C_0_ level at the primary screening. There is some evidence that PCD patients might be asymptomatic, such as the mothers of Families 1–6. Lacking of sufficient asymptomatic cases and follow-up, it is not yet clear whether potential health risks exist (12). A possible explanation for these asymptomatic cases might be that asymptomatic adult individuals may be unaware of their own defects. Fatty acid oxidation defects, such as medium chain acyl-CoA dehydrogenase deficiency, might not result in sudden death or other acute conditions unless the individual with the condition is under severe stress (28–30). Hence, it is crucial that asymptomatic PCD patients receive preventive treatments with carnitine supplementation for any potential decompensation attributed to intercurrent illness or stress.

In conclusion, the aim of the research was to determine if the application of MS/MS for neonatal screening combined with DNA analysis can diagnose PCD. Overall, newborn screening can result in the diagnosis of maternal PCD, and it is vital that low maternal C_0_ levels are evaluated at the secondary screening. PCD can be present at any age, with a wide phenotypic spectrum ranging from metabolic decompensation in infancy to an asymptomatic presentation in adulthood, or PCD may be asymptomatic but manifest or be exacerbated during pregnancy. To our knowledge, early universal L-carnitine treatment played a significant role in the favorable prognosis of patients with PCD. In addition, the current data highlights the importance of the diagnosis of PCD in neonatal screening. Because of the limited time, this study lacks more clinical cases, including PCD cases with exon skipping and deletions. Despite its exploratory nature, this study offers some insight into maternal PCD in newborn screening. Therefore, there is a definite need for additional research in the future.

## Ethics Statement

This study was approved by the Ethics Committee of Xuzhou Maternity and Child Health Care Hospital, and the individual written informed consents were obtained from the parents of the infants involved in this study.

## Author Contributions

WZ and MG: conception and design of study; WZ, HL, and YZ: acquisition of data; WZ, HL, TH, and CW: analysis and/or interpretation of data; WZ, HL, MG, and CW: drafting the manuscript; WZ, TH, and CW: revising the manuscript critically for important intellectual content.

### Conflict of Interest Statement

The authors declare that the research was conducted in the absence of any commercial or financial relationships that could be construed as a potential conflict of interest.
