# Supplementary figures and images for: Biochemical, Molecular, and Clinical Characterization of Patients With Primary Carnitine Deficiency via Large-Scale Newborn Screening in Xuzhou Area
Source: Front Pediatr. 2019 Feb 26;7:50. doi: 10.3389/fped.2019.00050 (PMC6399307; doi:10.3389/fped.2019.00050)

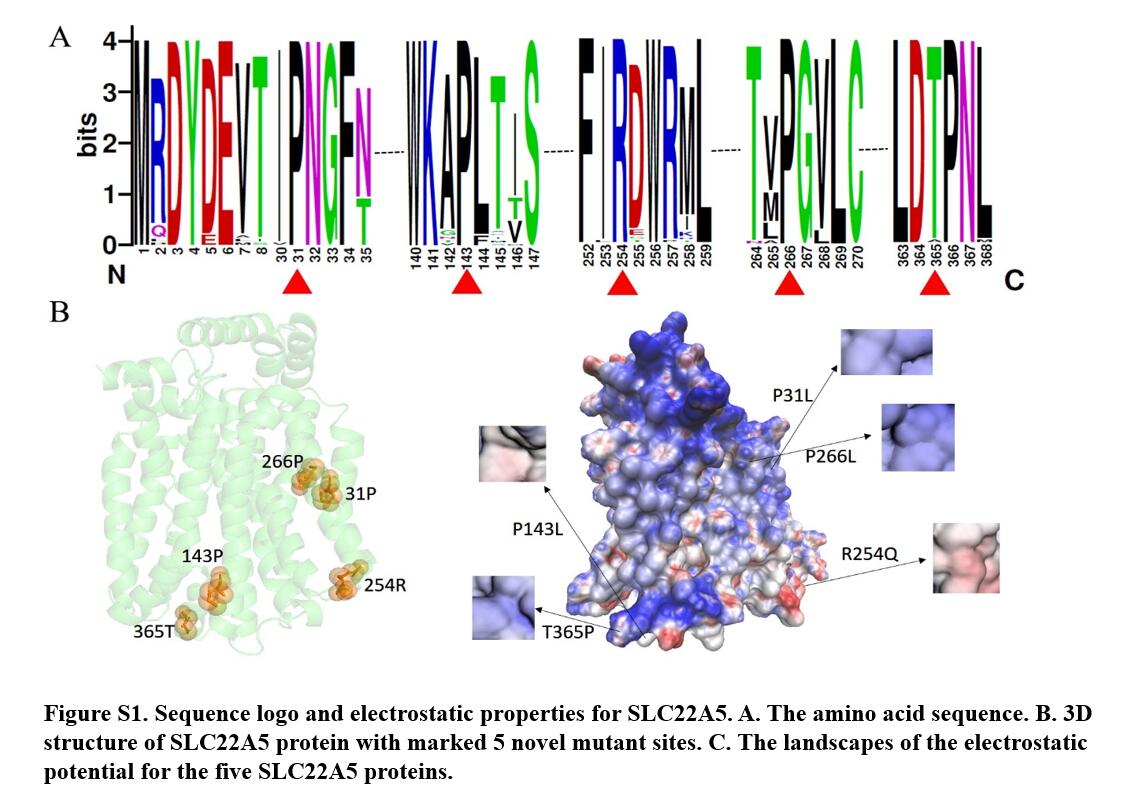

Supplement: Supplementary file 1 [file Image_1.JPEG]
